# Estimating COVID-19-Related Infections, Deaths, and Hospitalizations in Iran Under Different Physical Distancing and Isolation Scenarios

**DOI:** 10.34172/ijhpm.2020.134

**Published:** 2020-08-01

**Authors:** Hamid Sharifi, Yunes Jahani, Ali Mirzazadeh, Milad Ahmadi Gohari, Mehran Nakhaeizadeh, Mostafa Shokoohi, Sana Eybpoosh, Hamid Reza Tohidinik, Ehsan Mostafavi, Davood Khalili, Seyed Saeed Hashemi Nazari, Mohammad Karamouzian, Ali Akbar Haghdoost

**Affiliations:** ^1^HIV/STI Surveillance Research Center, and WHO Collaborating Center for HIV Surveillance, Institute for Futures Studies in Health, Kerman University of Medical Sciences, Kerman, Iran.; ^2^Department of Biostatistics and Epidemiology, School of Public Health, Kerman University of Medical Sciences, Kerman, Iran.; ^3^Modeling in Health Research Center, Institute for Futures Studies in Health, Kerman University of Medical Sciences, Kerman, Iran.; ^4^Department of Epidemiology and Biostatistics, Institute for Global Health Sciences, University of California San Francisco, San Francisco, CA, USA.; ^5^Dalla Lana School of Public Health, University of Toronto, Toronto, ON, Canada.; ^6^Department of Epidemiology and Biostatistics, Research Centre for Emerging and Reemerging Infectious Diseases, Pasteur Institute of Iran, Tehran, Iran.; ^7^Prevention of Metabolic Disorders Research Center, Research Institute for Endocrine Sciences, Shahid Beheshti University of Medical Sciences, Tehran, Iran.; ^8^Prevention of Cardiovascular Disease Research Center, Department of Epidemiology, School of Public Health and Safety, Shahid Beheshti University of Medical Sciences, Tehran, Iran.; ^9^School of Population and Public Health, Faculty of Medicine, University of British Columbia, Vancouver, BC, Canada.

**Keywords:** COVID-19, Modeling, Physical Distancing, Isolation, Iran

## Abstract

**Background:** Iran is one of the first few countries that was hit hard with the coronavirus disease 2019 (COVID-19) pandemic. We aimed to estimate the total number of COVID-19 related infections, deaths, and hospitalizations in Iran under different physical distancing and isolation scenarios.

**Methods:** We developed a susceptible-exposed-infected/infectious-recovered/removed (SEIR) model, parameterized to the COVID-19 pandemic in Iran. We used the model to quantify the magnitude of the outbreak in Iran and assess the effectiveness of isolation and physical distancing under five different scenarios (A: 0% isolation, through E: 40% isolation of all infected cases). We used Monte-Carlo simulation to calculate the 95% uncertainty intervals (UIs).

**Results:** Under scenario A, we estimated 5 196 000 (UI 1 753 000-10 220 000) infections to happen till mid-June with 966 000 (UI 467 800-1 702 000) hospitalizations and 111 000 (UI 53 400-200 000) deaths. Successful implantation of scenario E would reduce the number of infections by 90% (ie, 550 000) and change the epidemic peak from 66 000 on June 9, to 9400 on March 1, 2020. Scenario E also reduces the hospitalizations by 92% (ie, 74 500), and deaths by 93% (ie, 7800).

**Conclusion:** With no approved vaccination or therapy available, we found physical distancing and isolation that include public awareness and case-finding and isolation of 40% of infected people could reduce the burden of COVID-19 in Iran by 90% by mid-June.

## Background

**Key Messages**

**Implications for policy makers**

- With no interventions, over 5 million severe acute respiratory syndrome coronavirus 2 (SARS-CoV-2) infections, 1 million hospitalizations, up to 200 000 deaths could happen in Iran until mid-June.
- Case-finding and isolation of 40% of infected people can reduce the burden of coronavirus disease 2019 (COVID-19) in Iran by 90% by mid-June.
- It seems the burden of the epidemic in Iran is substantial, but Iran might have sufficient hospital bed capacity for COVID-19 based on the most probable scenarios.

**Implications for the public**

With no effective vaccination or treatment at hand, advocating and enforcing physical distancing and isolation along with public education on prevention measures could significantly reduce the burden of coronavirus disease 2019 (COVID-19) in Iran.

The coronavirus disease 2019 (COVID-19) was declared a pandemic on March 11, 2020, and the disease is has spread around the globe. The risk is relatively low for the general population, although people aged 65 years and over, those with suppressed immune systems, and people with underlying medical conditions (eg, cardiovascular or respiratory diseases) are at increased risk of adverse outcomes. The infection fatality rate (IFR) of the infection is estimated to be around 2% (95% CI: 2%-3%) and as of June 11, 2020, a total number of 7 480 281 confirmed cases, 3 794 453 recovered cases, and 419 472 deaths have been reported worldwide.^1^ Iran is one of the hardest hit countries by COVID-19 and has been struggling with controlling the disease for several months. The first confirmed cases of COVID-19-related deaths were reported on February 19 in the city of Qom; 200 km away from Iran’s capital city of Tehran. As of June 5, 2020, a total number of 169 425 confirmed cases, 132 038 recovered cases, and 8209 deaths have been reported and COVID-19 has spread to all of its provinces^2^; figures that are highest across the Eastern Mediterranean region.^3^

The susceptible-exposed-infected/infectious-recovered/ removed (SEIR) model provides a mathematical framework to explain the spread of infectious diseases and has been previously used for estimating the epidemiological parameters of several infectious diseases such as measles, Ebola, and influenza.^4-6^ SEIR could also help evaluate the impact of implementing various interventions (eg, isolation and physical distancing policies) aimed at controlling the pandemic growth and flattening the epidemic curve. Physical distancing control measures are policies that aim to minimize close contacts within communities and include individual-level (eg, quarantine, isolation) and community-level (eg, closure of educational and recreational settings, non-essential businesses, and cancellation of public/mass/crowded gatherings) approaches.^7,8^

**Figure 1:**
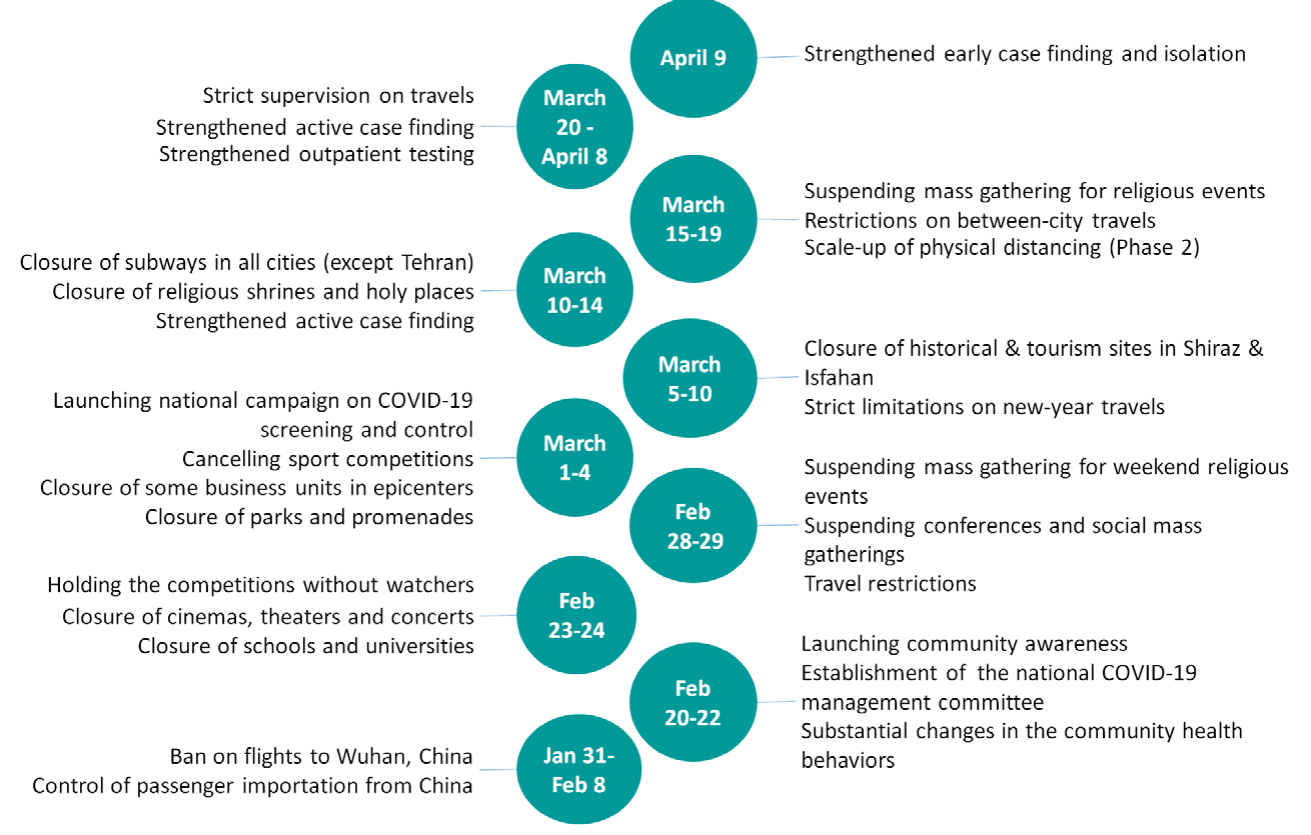
Iran’s Interventions Aimed at Controlling the COVID-19 Epidemic Since the Beginning of the Outbreak. Abbreviation: COVID-2019, coronavirus disease 2019.

In Iran, the physical distancing and isolation interventions were scaled up in late February and early March by nationwide closure of schools, cancellation of sports events and Friday or congregational prayers as well as the closure of all non-essential services, tourism sites, and shopping malls (Figure 1). Iran also closed its holy shrines in Mashhad and Qom in mid-March. Moreover, while there were no mandatory shelter-in-place or lockdown orders, people were encouraged to stay at home. People were also asked to avoid non-essential travels during the new year holidays (ie, Nowruz) from March 19 to 26; however, no restrictions for domestic or international travels were imposed.^9^ Despite implementing various physical distancing control measures, our understanding of their impact on the magnitude of COVID-19-related new infections, hospitalizations, and deaths remains limited. In this study, we aim to provide an estimate of these epidemiological parameters and approximate the peak date of the epidemic in Iran under different physical distancing and isolation scenarios. These estimates are of particular importance for COVID-19-related health policy, planning, and financing purposes in Iran.

## Methods

###  Model Description 

**Figure 2:**
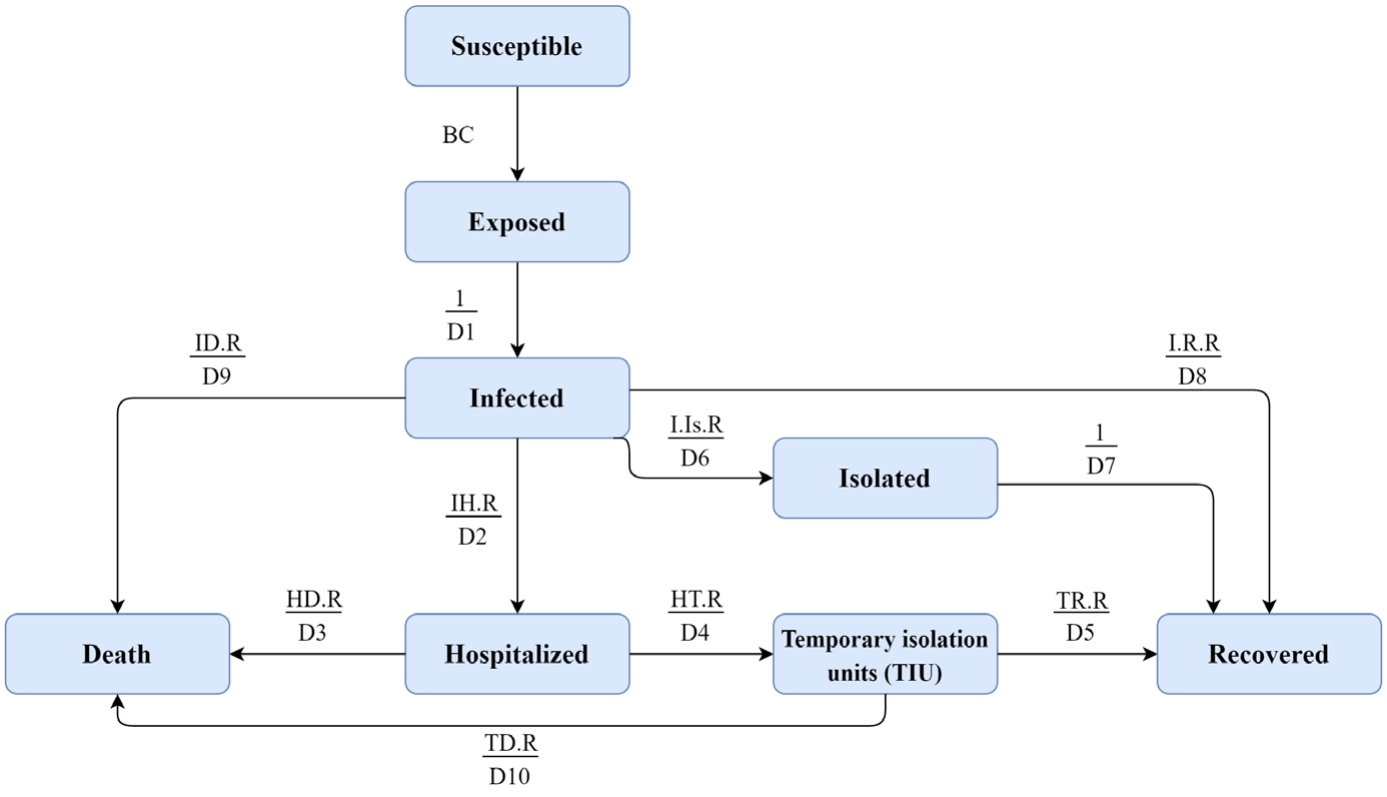
The SEIR Conceptual Model From Susceptible to Recovery or Deaths. Abbreviation: SEIR, susceptible-exposed-infected/infectious-recovered/removed.

We developed a compartmental model to estimate the total number of COVID-19 patients, hospitalizations and deaths in Iran as well as its capital city of Tehran (Figure 2). We used an extended SEIR model that divides the target populations (ie, Iran and Tehran as the capital) into different compartments. The conceptual framework of the COVID-19 transmission model is presented in Supplementary file 1. In brief, we considered the following compartments: (*a*) *susceptible*, referring to the total number of individuals (ie, hosts) who have been susceptible to COVID-19. We assumed the entire population as susceptible in our model; (*b*) *exposed*, referring to individuals who are exposed to COVID-19 while they are asymptomatic and not yet infectious; (*c*) *infected*, referring to infected people who demonstrate clinical symptoms after their incubation period and have the potential to transmit the disease to other susceptible individuals; and (*d*) *recovered/removed*, depending on the severity of the disease. We assumed that the infected people will (*i*) be recovered and immune from re-infection and therefore, no longer transmit the infection, (*ii*) have mild to moderate clinical symptoms and follow home-isolation guidelines without the need to be hospitalized, or (*iii*) have severe clinical symptoms and require hospitalization. These individuals would either be recovered or die and therefore, removed from the model. Monte Carlo method was used to estimate the 95% uncertainty intervals (UIs). To do this, we used the statistical distribution of a set of parameters obtained from both the existing evidence and expert opinion (Please see Table S1, Supplementary file 2). Data were analyzed using Vensim DSS 6.4E software.

###  Model Parameters and Calibrations 

Based on the country’s official reports and available epidemiological data, January 21, 2020 was considered as the initial day of the COVID-19 outbreak in Iran. We used several parameters as model inputs and obtained their values from a comprehensive literature review and published articles in relation to COVID-19, as well as relevant parameters derived from estimates in similar epidemics, in particular, H1N1 influenza.^10^ We first shared the initial values of the parameters with Iran’s national and scientific committees and experts, and made the necessary adjustments, accordingly. We compared the revised values of these parameters with the literature as well as the epidemic pattern in Iran. We then made the final revisions for the values of the parameters used as the extended SEIR model inputs. Please see Tables S1 and S2 in Supplementary file 2 for more details.

The impact of seasonality was considered in calculating the transmission probability (ie, beta coefficient) of the disease, indicating the potential for some level of change in transmissibility of the virus from one season to another.^10^ Therefore, we assumed that COVID-19 might behave the same as influenza where the transmission of the virus may be reduced by approaching warm seasons (ie, spring and summer). We then considered the end of December in winter with the highest transmission probability and the end of June in summer with the least transmission probability. The minimum and maximum values of the seasonal changes were considered to be 0.02 and 0.045, respectively. A time-varying state was considered for the effective contact rate(ie, parameter C). We first incorporated the value of 14 in model for Tehran and 13 in the national model in the early weeks of the epidemic.^11-15^ After the announcement of the epidemic by health officials, multiple public health measures were implemented as a response to the epidemic to reduce contact and transmission rates in the public. Approaching the assumed end of the epidemic, we considered the value of 5 for the contact rate parameter with some fluctuations due to Nowruz holidays which overlapped with this period. Please see Table S2 in Supplementary file 2 for more details. Five possible scenarios were considered for isolation of the infected cases (Table 1).

**Table 1:** Different Isolation Scenarios for Estimation of COVID-19-Related Infections, Hospitalizations, and Deaths in Iran

**Isolation Scenario**	**Description**
Scenario A	Isolation for the entire period of the epidemic was considered to be 0% (ie, worst-case scenario).
Scenario B	Prevention policies were encouraged and the overall mean of isolation for the entire period of the epidemic was considered to be 10%.
Scenario C	Isolation was considered to be 10% from January 21 to February 19, 15% after the initiation of the epidemic from February 20 to March 10, and 20% from March 11 to June 19, 2020. These policies correspond to the *minimal* possible interventions of the health system, behaviour change of the public, and containment strategies.
Scenario D	Isolation was considered to be 10% from January 21 to February 19, 15% after the initiation of the epidemic from February 20 to March 10, and finally 30% from March 11 to June 19, 2020, which are the results of the *moderate* possible interventions of the enhanced health system, social and behavioural change of the public (eg, physical distancing, hand washing), and containment strategies (eg, closing schools and universities).
Scenario E	Isolation was considered to be 10% from January 21 to February 19, 15% after the initiation of the epidemic from February 20 to March 10, and finally 40% from March 11 to June 19, 2020. These policies correspond to the *maximum* possible interventions of the health system, behaviour change of the public, and containment strategies.

Abbreviation: COVID-2019, coronavirus disease 2019.

###  Visual Calibration

 As the number of infections and hospitalizations were largely underestimated, we calibrated the models using daily reported deaths. Daily reported deaths were depicted in contrast to the number of estimated cases in each scenario.

###  Reproduction Number

Basic reproduction number (R_0_) defines the average number of secondary infections caused by each infected case in a population that all individuals are susceptible.^16,17^ Effective reproductive number (R_e_) is a particular threshold in epidemiology because the impact of interventions are shown by this index. We calculated the effective reproduction number (R_e_) in each stage using the generation matrix method.^18^ We presented how to calculate R_e_formulas in Supplementary file3.


$$R_{e}=\frac{\beta \left(t\right)*C\left(t\right)}{\left(\frac{I.Is.R}{D6}+\frac{I.R.R}{D8}+\frac{IH.R}{D2}+\frac{ID.R}{D9}\right)}$$

###  Infection Fatality Rate 

The IFR was calculated using this formula^19,20^:


$$IFR=\frac{Number\;of\;deaths\;due\;to\;COVID-19}{Total\;number\;of\;actual\;\inf ection\;with\;COVID-19}*100$$

## Results

###  Validation of the Model

**Figure 3:**
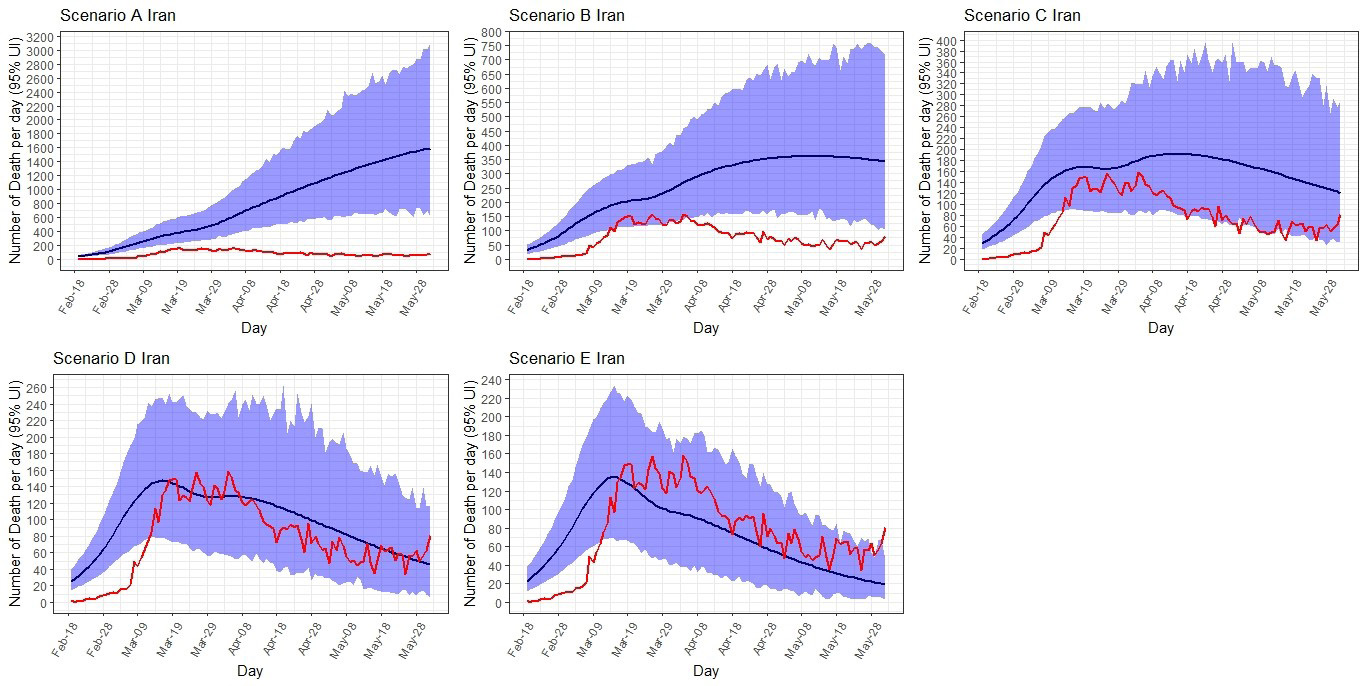
Daily Observed Deaths in Comparison of the Reported Deaths in the National Model in 5 Scenarios. The blue area shows UIs for the estimated deaths. Abbreviation: UI, uncertainty interval.

As seen in Figure 3, the observed deaths are close to the estimated cases in scenario C, D, and E. As the sensitivity of the disease surveillance systems across the country were low at the begging of the epidemic and during the first three weeks, the model predicted higher number of deaths than the official reports between 19 February to 10 March.

###  Expected Number of Infected Cases 

*Iran: *Under scenario A, the epidemic would peak on June 9 at around 66 000 (95% UI: 15 600-145 000) new infected cases per day. Total number of infected cases by June 19 is expected to be 5 196 000 (95% UI: 1 753 000-10 220 000). Under scenario B, the peak would occur on April 30, 2020 at around 16 500 (95% UI: 5400-35 000) infected cases a day; a total of 1 643 300 (95% UI: 603 400-3 380 000) infections would occur by June 19, 2020. Under scenario C, the peak would occur on 31 March at around 10 700 (95% UI: 4600-20 000) infected cases a day; a total of 949 600 (95% UI: 368 000-1 960 000) infections would occur by June 19, 2020. The number of infected cases at the peak time under scenario D (9400 cases on March 1) and scenario E (9400 cases on March 1) are decreased further. The total number of infected cases by June 19, 2020 under scenario D (689 000 cases on March 1, 2020) and scenario E (550 000 cases on March 1, 2020) are decreased further.

**Figure 4:**
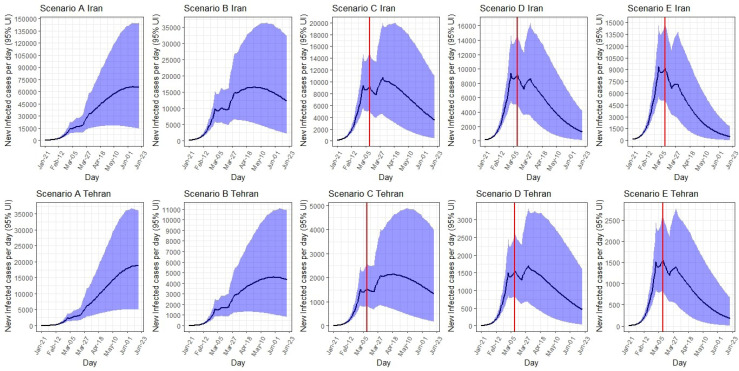
The Estimated Number of New Infected Cases Per Day in Iran and Tehran Under 5 Different Isolation Scenarios From January 21, 2020 to June 19, 2020. The red line refers to the time when interventions were implemented and the blue area corresponds to UIs. Abbreviation: UI, uncertainty interval.

*Tehran:* Under scenario A, the number of new cases per day in Tehran would peak on June 19, 2020 at around 19 000 (95% UI: 5000-36 000). Total number of infected cases by June 19 is expected to be 1 228 000 (95% UI: 416 400 to 2 407 000). The number of new cases at peak and total number of cases decreased to lowest number from scenario B to E. Under scenario E, the number of new cases per day would reduce to 1600 (95% UI: 900-2600), and the total number of infected cases by June 19 would reduce to 106 400 (95% UI: 41 200-225 000) **(**Table 2, Figure 4).

**Table 2:** The Estimated Date for Epidemic Peak, Number of Infected, Hospitalized, and Deceased Patients in Iran and Tehran Under 5 Different Scenarios From January 21, to June 19, 2020

	**Scenario A**		**Scenario B**		**Scenario C**		**Scenario D**		**Scenario E**	
	**Iran**	**Tehran**	**Iran**	**Tehran**	**Iran**	**Tehran**	**Iran**	**Tehran**	**Iran**	**Tehran**
**Infected Cases**										
Peak date	9-Jun	19-Jun	30-Apr	30-May	31-Mar	20-Apr	1-Mar	31-Mar	1-Mar	11-Mar
Number (95% UI) of new cases per day on peak	66 000 (15 600, 145 000)	19 000 (5000, 36 000)	16 500 (5400, 35 000)	4600 (1000, 11 000)	10 700 (4600, 20 000)	2200 (700, 4700)	9400 (5600, 15 000)	1700 (700, 3300)	9400 (5600, 15 000)	1600 (900, 2600)
Total number (95% UI) until June 19, 2020	5 196 000 (1 753 000, 10 220 000)	1 228 000 (416 400, 2 407 000)	1 643 300 (603 400, 3 380 000)	398 600 (131 000, 852 000)	949 600 (368 000, 1 960 000)	211 800 (71 700, 467 700)	689 000 (286 000, 1 363 000)	142 000 (51 200, 309 800)	550 000 (243 500, 1 070 200)	106 400 (41 200, 225 000)
**Hospitalized Cases**										
Peak date	19-Jun	19-Jun	12-May	10-Jun	14-Apr	28-Apr	15-Mar	10-Apr	14-Mar	15-Mar
Number (95% UI) of the existing cases on peak	67 800 (25 100, 131 000)	19 000 (7800, 34 200)	14 600 (5800, 28 100)	4000 (1200, 8600)	7800 (3200, 14 500)	1600 (550, 3400)	6600 (3500, 10 900)	1100 (400, 2100)	6500 (3400, 10 600)	1000 (500, 1800)
Total number (95% UI) until June 19, 2020	966 000 (467 800, 1 702 000)	228 000 (108 800, 400 800)	279 500 (119 200, 527 700)	65 500 (26 100, 127 700)	147 000 (56 200, 292 300)	32 000 (11 000, 67 700)	98 700 (38 100, 195 600)	20 000 (6700, 42 700)	74 500 (29 400, 146 500)	14 000 (4900, 29 700)
**Deaths**										
Total number (95% UI) until June 19, 2020	111 000 (53 400, 200 000)	25 800 (12 000, 47 000)	32 900 (14 700, 62 000)	7600 (3100, 15 000)	17 700 (7300, 34 400)	3800 (1400, 7900)	11 000 (4500, 21 700)	2200 (800, 4600)	7800 (3200, 15 000)	1500 (500, 3000)
Total IFR	2.2%	2.1%	2.0%	1.9%	1.9%	1.8%	1.6%	1.5%	1.4%	1.3%

Abbreviations: UI, Uncertainty Interval; IFR, infection fatality rate.

###  Expected Number of Hospitalized Cases 

*Iran:* Under scenario A, the number of hospitalized cases would peak on June 19 at around 67 800 (95% UI: 25 100- 131 000) per day. Total number of hospitalized cases by June 19 is expected to be 966 000 (95% UI: 467 800-1 702 000). Under scenario B, the peak would occur on May 12 at around 14 600 hospitalized cases a day. Under scenario C, the peak would occur on April 14 at around 7800 hospitalized cases a day. The number of hospitalized cases at the peak time under scenario D was 6600 cases (on March 15) and under scenario E was 6500 cases (on March 14).

**Figure 5:**
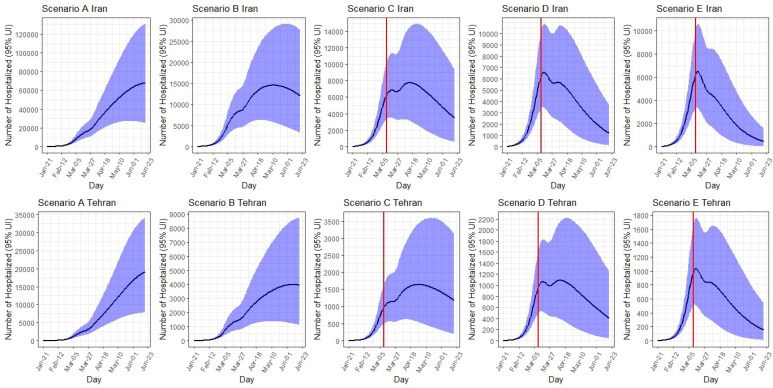
The Estimated Number of Existing Hospitalized Cases in Iran and Tehran Under 5 Different Isolation Scenarios From January 21, 2020 to June 19, 2020. The red line refers to the time when interventions were implemented and the blue area corresponds to UIs. Abbreviation: UI, uncertainty interval.

*Tehran: *Under scenario A, the number of hospitalized cases per day in Tehran would peak on June 19 at around 19 000 (95% UI: 7800-34 200). Total number of hospitalized cases by June 19 is expected to be 228 000 (95% UI: 108 800-400 800). The number of hospitalized cases at peak and total number of hospitalized cases are decreased to lowest number from scenario B to E. Under scenario E, the number of hospitalized cases per day decreases to 1000 (95% UI: 500-1800), and the total number of hospitalized cases by June 19 decreases to 14 000 (95% UI: 4900-29 700) (Table 2, Figure 5).

###  Expected Number of Deaths 

*Iran:* Up to June 19, the total expected number of deaths would range from 111 000 (95% UI: 53 400 to 200 000) under scenario A to 7800 (95% UI: 3200-15 000) under scenario E. The corresponding IFR under scenario A is 2.2%, which would decrease to 1.4% under scenario E.

**Figure 6:**
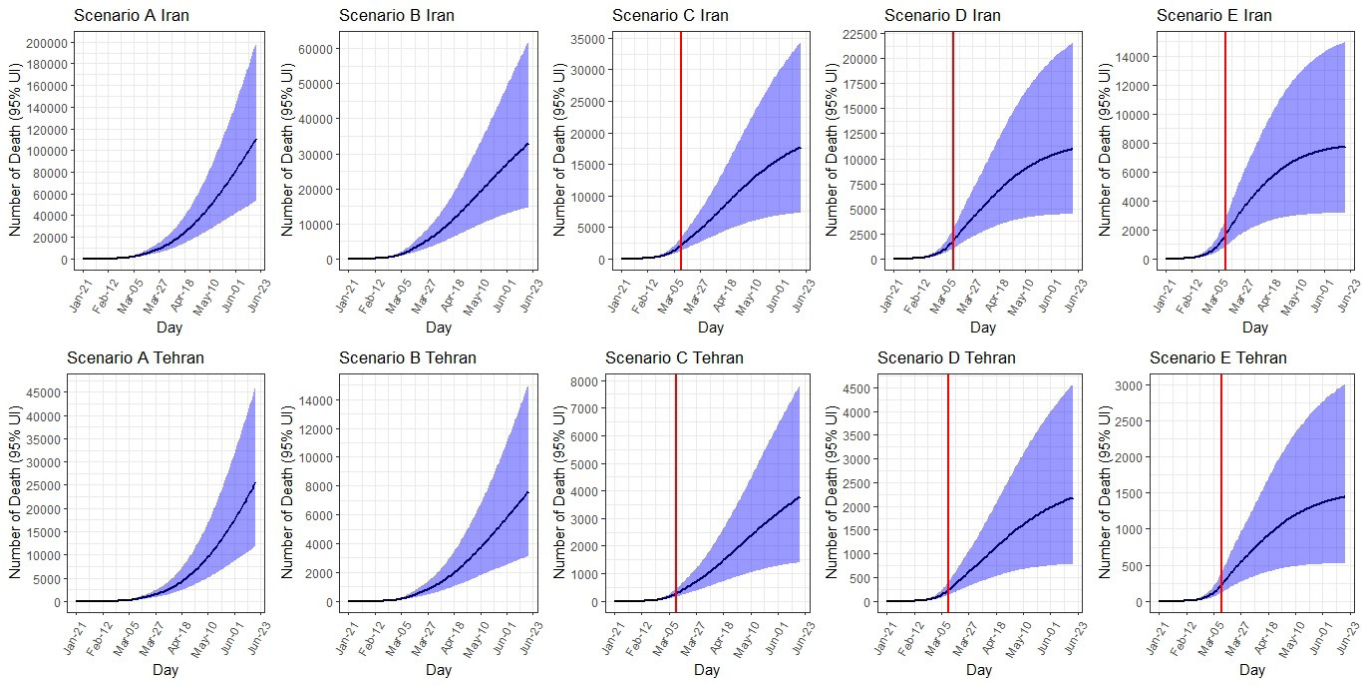
The Estimated Number of Deaths in Iran and Tehran Under 5 Different Isolation Scenarios From January 21, 2020 to June 19, 2020. The red line refers to the time when interventions were implemented and the blue area corresponds to UIs. Abbreviation: UI, uncertainty interval.

*Tehran: *Up to June 19, the total expected number of deaths would range from 25 800 (95% UI: 12 000 to 47 000) under scenario A to 1500 (95% UI: 500 to 3000) under scenario E. The corresponding IFR under scenario A is 2.1%, which would decrease to 1.3% under scenario E (Table 2, Figure 6).

###  Reproduction Number (R)

**Figure 7:**
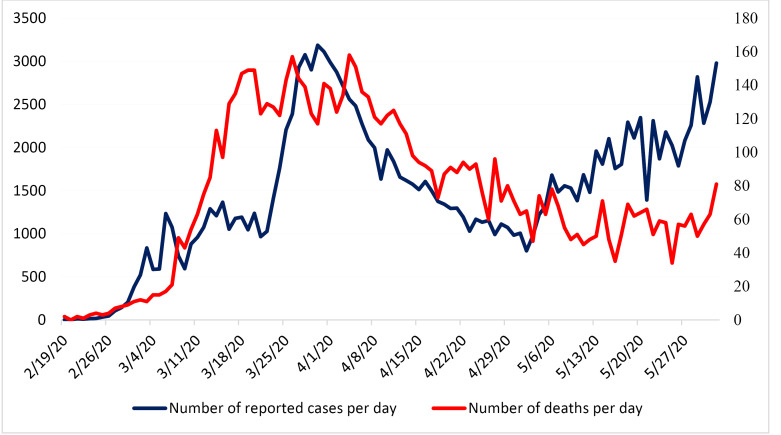
The Number of Reported Cases and Deaths Per Day From the Begging of the Epidemic to June 1, 2020 in Iran.

At the begging of the epidemic, the R_e_ was expectedly higher (ie, 4.70 for Tehran and 4.40 for Iran in scenario A to 3.93 in Tehran and 3.64 for Iran in scenarios B-E). The R_e_ decreased slightly by the end of the modeling process. From April 1 to July 19, the reproduction number was calculated as 1.18 in scenario A to 0.64 in scenario E (Table 3, Figure 7).

**Table 3:** Reproduction Number of the Virus in Tehran and Iran Based on Different Isolation Scenarios From January 21, 2020 to July 19, 2020

**Time Intervals**	**Scenario**	** R**_e_ ** for Tehran**	**R**_e_ ** for Iran**
Jan 21, 2020 to Jan 30, 2020	A	4.70	4.40
	B	3.93	3.64
	C	3.93	3.64
	D	3.93	3.64
	E	3.93	3.64
Jan 31, 2020 to Feb 9, 2020	A	4.31	3.97
	B	3.56	3.29
	C	3.56	3.29
	D	3.56	3.29
	E	3.56	3.29
Feb 10, 2020 to Feb 19, 2020	A	3.88	3.56
	B	3.22	2.95
	C	3.22	2.95
	D	3.22	2.95
	E	3.22	2.95
Feb 20, 2020 to Feb 29, 2020	A	3.16	2.84
	B	2.39	2.15
	C	2.39	2.15
	D	2.39	2.15
	E	2.39	2.15
Mar 1, 2020 to Mar 10, 2020	A	2.31	2.31
	B	1.15	1.15
	C	1.15	1.15
	D	1.15	1.15
	E	1.15	1.15
Mar 10, 2020 to Mar 20, 2020	A	1.46	1.46
	B	1.21	1.21
	C	1.03	1.03
	D	0.89	0.89
	E	0.79	0.79
Mar 21, 2020, to Mar 31, 2020	A	1.68	1.68
	B	1.39	1.39
	C	1.18	1.18
	D	1.03	1.03
	E	0.91	0.91
Apr 1, 2020 to June 19, 2020	A	1.18	1.18
	B	0.98	0.98
	C	0.84	0.84
	D	0.73	0.73
	E	0.64	0.64

## Discussion

 Our results suggested that with no intervention (ie, scenario A), more than 5 million SARS-CoV-2 (severe acute respiratory syndrome coronavirus 2) infections (ie, 6.3% of total population) would occur in Iran till mid-June, of whom 18.9% would be hospitalized and 2.1% would die. However, under the best-case scenario (ie, scenario E), the number of infected patients, hospitalizations, and deaths could be reduced by 90%, 92%, and 93%, respectively. Our projection in scenario C, which is a middle-ground scenario, appeared to be aligned with the current national trend in reported cases. Even under scenario C, the burden of the epidemic in Iran will be large, last for several months, yet might not surpass the healthcare system’s capacity.

Based on the reported deaths and peak date for the number of confirmed cases per day, scenarios C or D are the most plausible ones for COVID-19 outbreak evolvement in Iran. Under scenario C, 10 100 new infections would have occurred on April 5, 2020, which is four times the number of confirmed cases on that date. This scenario expects the total number of infections and deaths by mid-June to be around 950 000 and 17 600, respectively; more than two times the number of observed deaths to date (ie, 8209 cases by June 6, 2020). Our findings however, suggest that scenario B is the most plausible scenario in the capital city of Tehran which highlights the importance of implementing special measures and policies in this city to reduce the spread of the disease. Our findings in scenario B are comparable with other dynamic model estimations in Iran,^21^ that have predicted around 1.6 million (90% CI, 0.9 million-2.6 million) cases, and 58 000 (90% CI, 32 000-97 000) deaths under their most optimistic scenarios. The above-mentioned model however, had three structural assumptions on three major parameters (ie, duration of illness as 14 days, asymptomatic period as 4 days, and transmission probability as 0.25 during the asymptomatic period) and did not account for seasonality distribution.^21^

 Our model indicated that under scenario C (ie, the best fitted trend with reported cases/deaths), Iran might have sufficient hospital bed capacity for COVID-19. As of April 8, 2020, public hospitals in Iran, which are mainly responsible for the COVID-19 response, had 146 137 beds (9134 in intensive care units [ICU] and 9730 ventilators). By discharging inpatient cases and postponing elective medical care and surgeries, Iran has currently allocated 100 437 empty beds, 5790 empty ICU beds, and 4650 ventilators towards COVID-19 patients. This might be sufficient for sever COVID-19 patients as predicted by scenario C which would still require appropriate supplies and staffing. As of April 8, 2020, there were 0.41 physician and 1.14 nurse per public hospital bed in Iran. Even under scenario C, the shortage of healthcare workers would be challenging, although there are sufficient beds and ventilators.

Our model showed that non-pharmaceutical innervations actually worked to control the epidemic in Iran. In addition to individual- and community-level physical distancing control measures that have been implemented in Iran from late February to late March, the number of testing for COVID-19 screening was also increased from 1000 tests per day on March 12, 2020 to about 20 000 tests per day on May 27, 2020. This has led to an increase in case finding, contact tracing of new cases, and isolation of confirmed cases. Despite the persistent increase in testing, the number of confirmed cases per day peaked at 3200 on March 30, 2020 and then decreased to 1374 cases per day on April 18, 2020.^22^ Moreover, measuring the real intensity and coverage of physical distancing in Iran is challenging as these practices have not been closely followed during to the new year (ie, Nowruz) holidays between March 19 and 26, 2020.^23,24^ Such a significant increase in travels made the government to enforce physical distancing by lockdown, and closing the roads. While we did account for the Nowruz effect in our model by increasing the number of contacts per day (parameter C) from 5 to 6 from March 21 to March 31, 2020, the true effect of Nowruz on the current and future projected numbers remains unknown.

Our projections of the COVID-19 morbidity and mortality in Iran under different physical distancing and isolation scenarios are insightful for government’s policies regarding relaxing the physical distancing and isolation interventions. Although the decision on the timing of and approaches towards lifting the physical distancing restrictions is extremely difficult and varies across countries with different economic and healthcare infrastructures, it is critical to follow an evidence-informed approach to avoid the second and further waves of COVID-19 epidemics in Iran. A modeling study from China for example,^25^ showed that a stepwise (25% of the workforce working in weeks 1 to 2, 50% of the workforce working in weeks 3 to 4, and 100% of the workforce working and school resuming from week 5 forward) return to work or school at the beginning of April (about 5 months after the first case reported from China), is much more effective than the beginning of March. This study estimated that just a one-month delay in the stepwise lifting of the physical distancing measures would reduce the magnitude (92% by mid-2020, 24% by end-2020) the epidemic and delay its peak by two months and therefore avoid overwhelming the healthcare systems.^25^

In Iran, the daily number of cases declined for a few weeks in April, but the second wave of the epidemic is emerging. A recent executive order from the Iranian government lifted the restrictions on nationwide business shutdown and allowed most people to return to work only 2.5 months after the identification of the first COVID-19 case in Iran.^9,26^ The government has also planned to reopen schools in low-risk cities and non-essential low or medium-risk jobs (eg, all production units in industrial, business, technical service, and distributional sections), as well as removing the shelter-in-place order and resuming domestic and international travels. These decisions are mainly derived from the Iranian government’s economic challenges that have been elevated by the comprehensive sanctions imposed by the United States.^27,28^ While these concerns are understandable and longer shutdown of an already overstretched economy is a tough decision and would be very taxing on the government and the public, our findings as well as lessons learned from China,^25,29,30^ suggest that this approach might not be justified by evidence and would most likely risk overwhelming the healthcare systems with the next waves of COVID-19 epidemic in Iran; costs that might surpass the marginal economic benefits of opening businesses and schools a few months sooner. Nonetheless, it is fortunate that the Iranian Center for Disease Control is now planning to shift from physical distancing to targeted case-finding, intensify contact tracing and careful isolation of identified cases as well as self-quarantine of symptomatic people.

 Our study had three major limitations. First, some of the key parameters (eg, hospitalization rate, incubation period, transmission probability) that were used in the model were from other countries or expert opinion as empirical data from Iran were unavailable. To address this limitation, we reported a range of UIs. Second, the UIs are fairly wide for most of our results which resulted from the uncertainty in model parameters. Third, our projection for the course of the epidemic in the coming months in Iran is based on the assumption we made about implementing and sustaining the physical distancing as planned, and any changes in policy and public interventions may change the course of the epidemic. Despite these limitations, our results under different scenarios provide a foundation to measure the effect of interventions that are ongoing in the country.

## Conclusion

 With no available vaccination, prophylaxis or therapy, we found physical distancing and isolation that includes public awareness and case finding and isolation of 40% of the infected cases could reduce the burden of COVID-19 in Iran by 90% by mid-June. The IFR for the national model ranged from 1.4%-2.2%.

## Ethical issues

 The proposal of the present study was approved by ethics committee of Kerman University of Medical Sciences, Kerman, Iran (reference 98001239).

## Competing interests

 The senior author, AAH is the Deputy Minister of Education and the Head of National Committee on COVID-19 Epidemiology. The rest of the authors declare no conflict of interest, real or perceived.

## Authors’ contributions

 In this work HS, YJ, AM, MAG, and MN took the lead, were responsible for the data analysis and made substantial contributions to conception, design, and writing. MS, SE, HRT, and MK made substantial contributions to conception, design, and writing. EM, DK, and SSHN revised the study critically and contributed to conception. AAH revised the study critically, contributed substantially to conception, design, the interpretation of data, and drafting of the article.

## Funding

 The study did not receive any funding from any organization.

## Authors’ affiliations

^1^HIV/STI Surveillance Research Center, and WHO Collaborating Center for HIV Surveillance, Institute for Futures Studies in Health, Kerman University of Medical Sciences, Kerman, Iran. ^2^Department of Biostatistics and Epidemiology, School of Public Health, Kerman University of Medical Sciences, Kerman, Iran. ^3^Modeling in Health Research Center, Institute for Futures Studies in Health, Kerman University of Medical Sciences, Kerman, Iran. ^4^Department of Epidemiology and Biostatistics, Institute for Global Health Sciences, University of California San Francisco, San Francisco, CA, USA. ^5^Dalla Lana School of Public Health, University of Toronto, Toronto, ON, Canada. ^6^Department of Epidemiology and Biostatistics, Research Centre for Emerging and Reemerging Infectious Diseases, Pasteur Institute of Iran, Tehran, Iran. ^7^Prevention of Metabolic Disorders Research Center, Research Institute for Endocrine Sciences, Shahid Beheshti University of Medical Sciences, Tehran, Iran. ^8^Prevention of Cardiovascular Disease Research Center, Department of Epidemiology, School of Public Health and Safety, Shahid Beheshti University of Medical Sciences, Tehran, Iran. ^9^School of Population and Public Health, Faculty of Medicine, University of British Columbia, Vancouver, BC, Canada.

## Supplementary files


Supplementary file 1. The SEIR Model.

Supplementary file 2 contains Tables S1 and S2.

Supplementary file 3. Calculation of Reproduction Number (R_0_).
