# Supplementary material for: Estimating COVID-19-Related Infections, Deaths, and Hospitalizations in Iran Under Different Physical Distancing and Isolation Scenarios
Source: Int J Health Policy Manag. 2020 Aug 1;11(3):334–43. doi: 10.34172/ijhpm.2020.134 (PMC9278464; doi:10.34172/ijhpm.2020.134)
Supplement: Supplementary file 1 — The SEIR Model. [file ijhpm-11-334-s001.pdf]

Supplementary file 1. The Susceptible-Exposed-Infected/Infectious-Recovered/Removed (SEIR) model

A. Supplementary Figure 1: The SEIR conceptual model

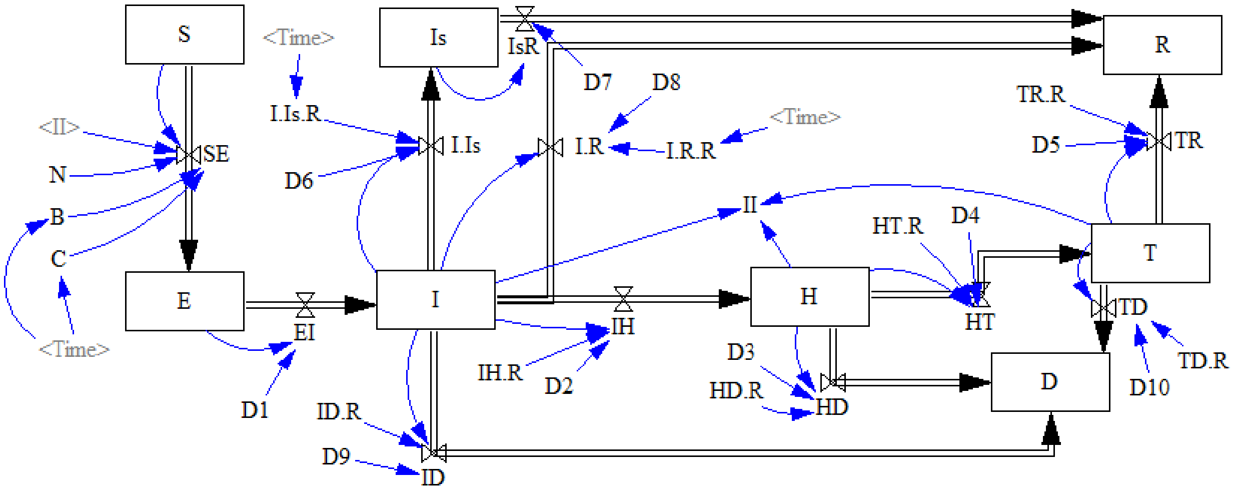

B. Model formation

As mentioned in the body of the manuscript, we used an extended form of the susceptible-exposed-infected/infectious-recovered/removed (SEIR) model, which is a dynamic modelling approach. Such epidemiological models are assumed to be compartmental; the target population will be divided into different sections or compartments. The conceptual framework of our model is shown above in section A. Using this model, we divided the population size of the target populations into the following compartments:

a) Individuals who are *susceptible* (S(t)): an individual (ie, host) is initially assumed to be susceptible to the virus (ie, COVID-19 here), and that the virus can be transmitted from infected individuals to susceptible individuals. In this model, the entire population of the target populations were considered to be susceptible. The differential equation of this compartment is shown in Equation 1:

Eq. 1 $$\frac{dS(t)}{dt} = -\beta(t)C(t)\frac{II(t)}{N}S(t)$$

where β(t) indicates transmissibility of the virus, and C(t) indicates the contact rates, and II(t) refers to the total number of infected people who transmit the infection, calculated as Infect (t)+(0.1×Temporary Isolation Units)+(0.02×Hospital) (explained below).

b) Individuals who are *exposed* (E(t)): refers to individuals who are exposed to the infection, but they are not yet infectious. These individuals are asymptomatic in this period. The differential equation of this compartment is shown in Equation 2:

Eq. 2 $$\frac{dE(t)}{dt} = \beta(t)C(t)\frac{II(t)}{N}S(t) - \frac{1}{D1}E(t)$$

c) Those who are *infected* (I(t)): in this model, infected patients after a period of time will demonstrate clinical symptoms of the infection and will transmit the infection to any other susceptible individuals. Equation 3 shows the differential equation of this compartment:

Eq. 3 $$\frac{dI(t)}{dt} = \frac{1}{D1}E(t) - \left(\frac{I.Is.R}{D6} + \frac{I.R.R}{D8} + \frac{IH.R}{D2} + \frac{ID.R}{D9}\right)I(t)$$

d) Those who *recovered*: depending on the severity of the infection, infected individuals will end up with one of the following four states:

i) Infected individuals who are recovered (R(t)), who will be assumed to be immune from re-infection and no longer transmit the infection (recovered box in Fig 1). Equation 4 shows the differential equation of this section:

Eq. 4 $$\frac{dR(t)}{dt} = \frac{TR.R}{D5}T(t) + \frac{I.R.R}{D8}I(t) + \frac{1}{D7}Is(t)$$

ii) Infected individuals who will have mild to moderate clinical symptoms, but they will be home-isolated without requiring hospitalization (IS(t)), and they will be recovered (isolated box in Fig 1). Equation 5 shows the differential equation of this section:

Eq. 5 $$\frac{dIs(t)}{dt} = \frac{I.Is.R}{D6}I(t) - \frac{1}{D7}Is(t)$$

iii) Infected individuals who will have severe clinical symptoms requiring hospitalization (hospitalized box in Fig 1). These individuals will have two possible outcomes: i) some hospitalized cases will be recovered and then discharged (T box in Fig 1), or ii) some will not respond to the medical care and die (death box in Fig 1). Equations 6 and 7 show the differential equation of hospital box (H(t)) and T box (T(t)) respectively:

Eq. 6 $$\frac{dH(t)}{dt} = \frac{IH.R}{D2}I(t) - (\frac{HD.R}{D3} + \frac{HT.R}{D4})H(t)$$

Eq. 7 $$\frac{dT(t)}{dt} = \frac{HT.R}{D4}H(t) - (\frac{TR.R}{D5} + \frac{TD.R}{D10})T(t)$$

iv) Infected individuals who will die (removed) without recovery (D(t)) (move to death box). Equation 8 shows the differential equation of this section:

Eq. 8 $$\frac{dD(t)}{dt} = \frac{\mathrm{HD.R}}{\mathrm{D3}}\mathrm{H(t)} + \frac{\mathrm{ID.R}}{\mathrm{D9}}\mathrm{I(t)} + \frac{TD.R}{D10}T(t)$$
