# Supplementary material for: Estimating COVID-19-Related Infections, Deaths, and Hospitalizations in Iran Under Different Physical Distancing and Isolation Scenarios
Source: Int J Health Policy Manag. 2020 Aug 1;11(3):334–43. doi: 10.34172/ijhpm.2020.134 (PMC9278464; doi:10.34172/ijhpm.2020.134)
Supplement: Supplementary file 2 — contains Tables S1 and S2. [file ijhpm-11-334-s002.pdf]

Supplementary file 2

Table S1. Disease and demographic inputs and parameters used in the SEIR model

Parameters	Definition	Values	References
Susceptible S(t)	Susceptible individuals to getting the infection at any time	At time 0 (start of the epidemic, January 21) n = 10 million for Tehran (capital) n = 83 million for the national model	[1,2]
Exposed E(t)	Exposed individuals to the virus at any time	At time 0 (start of the epidemic, January 21) n = 70 in Tehran n = 1170 in the national model	--
Infected I(t)	Infected individuals at any time	At time 0 n = 5 in Tehran n = 90 in the national model	--
Isolated Is(t)	Individuals who become home-isolated after getting the infection without hospitalization	At time 0 Value = 0	--
Recovered R(t)	Individuals who are recovered from the infection	At time 0 Value = 0	--
Hospital H(t)	Individuals who are hospitalized	At time 0 Value = 0	--
Death D(t)	Individuals who died of the infection	At time 0 Value = 0	--
Temporary Isolation Units T(t)	TIU individuals who are discharged from hospitals	At time 0 Value = 0	--
N	Country total population	N = 10 million for Tehran (capital) N = 83 million for the national model	[1,2]
C	The average number of contacts for each uninfected individual	The values of these parameters are	--

Parameters	Definition	Values	References
		reported in Appendix B Table 2.	
B (Beta)	Transmission probability (the probability of getting the infection when an infected individual contact with an uninfected individual)	Ranged from 0.0423 in 21 January to 0.025813 in 19 June Seasonality distribution: (((Sin (2 × 3.14 × (Time + 110) / 365)) + 1) × ((0.045 – 0.02) / 2)) + 0.02	[3–5]
II(t)	Infected individuals with the potential to infect uninfected people	I+(0.1×T)+(0.02×H)	--
SE	The average number of people from susceptible individuals are added to exposed individuals (per day)	B×C× (II/N)× S	--
EI	The average number of people from exposed individuals are added to infected individuals (per day)	E×(1/D1)	--
D1	The average duration taken for an exposed individual becomes an infected individual	Normal (5.33, 0.445)[a]	[6]
I.Is	The number of people who become home-isolated (per day)	(I)×(I.Is.R)×(1/D6)	--
D6	The average duration taken for an infected individual becomes home-isolated	Normal (3, 0.5)[a]	Expert Opinion
I.Is.R	% of infected individuals becomes isolated	According to the possible scenarios in the text	--
IsR	The number of people who become recovered after isolation (per day)	(Is)/D7	--
D7	The duration taken for an isolated individual becomes recovered	Normal (7.91,0.5)[a]	[6]
I.R	The total number of infected cases who become recovered (without isolation and without hospitalization)	(I)×(I.R.R)×(1/D8)	--

Parameters	Definition	Values	References
I.R.R	% of infected cases who become recovered (without isolation and without hospitalization)	According to the possible scenarios	--
D8	The average duration taken for an infected case becomes recovered (without isolation and without hospitalization)	Normal (10.91, 0.50)[a]	[6]
IH	Number of infected individuals who are hospitalized (per day)	I× (1/D2)×(IH.R)	--
IH.R	% of infected individuals who are hospitalized	Normal (0.05, 0.01)[a]	National Data and Expert Opinion
D2	The average duration taken for an infected individual stays in hospital	Normal (2, 0.5)[a]	Expert Opinion
HT	Number of hospitalized cases who are discharged from hospital (per day)	(HT.R)×(H)×(1/D4)	--
HT.R	% of hospitalized cases who discharged from hospital	Normal (0.9, 0.01)[a]	National Data and Expert Opinion
D4	The average duration taken for a hospitalized individual to be discharged	Normal (5, 0.5)[a]	National Data and Expert Opinion
TR	Number of individuals who are on recovery after discharge	(T)×(TR.R)×(1/D5)	--
TR.R	% of infected cases who recovered after discharge	N(0.995, 0.001)[a]	National Data and Expert Opinion
D5	The average duration taken to be recovered after discharge	Normal (7, 0.5)[a]	National Data and Expert Opinion
HD	Number of hospitalized, infected cases who die in hospital (per day)	(H)×(1/D3)×(HD.R)	--
HD.R	% of hospitalized, infected cases who die in hospital	1 – HT.R	--
D3	The average duration taken for a hospitalized, infected case to die in hospital	Normal (5, 0.5)[a]	Expert Opinion

Parameters	Definition	Values	References
ID	Number of infected cases who die from the infection without being hospitalized	(I)×(ID.R)×(1/D9)	--
ID.R	% of infected cases who die from the infection without being hospitalized	scenario A,B and C: 0.005<br>scenario D: 0.002<br>scenario E: 0	Expert Opinion
D9	The average duration taken to be died from the infection for infected individuals without being hospitalized	Normal (11, 0.50)[a]	[7] justify with expert opinion
TD	Number of infected cases who die after discharge (per day)	(T)×(TD.R)×(1/D10)	--
TD.R	% of infected cases who die from the infection after discharge (ie, 5 deaths in 1000 discharged cases)	N(0.005, 0.001)[a]	National Data<br>Expert Opinion
D10	The average duration taken to be died after discharge	Normal (7, 0.50)[a]	Expert Opinion

[a] Normal refers to the normal distribution (mean, SD)

Table S2. The values considered for effective contact rate (ie, C) within four months of the COVID-19 epidemic stratified by Tehran and national models

Months and 10-day intervals	**Contact rate (C)**		**Reference**
	**Tehran model**	**National model**	
From Jan 21, 2020, to Jan 30, 2020	14	13	[7–11]
From Jan 31, 2002, to Feb 9, 2020	13	12	
From Feb 10, 2020, to Feb 19, 2020	12	11	
From Feb 20, 2020, to Feb 29, 2020	10	9	
From Mar 1, 2020, to Mar 20, 2020	5	5	
From Mar 21, 2020, to Mar 31, 2020	6*	6*	
From Apr 1, 2020, to June 19, 2020	5	5	

* Contact rates were assumed to increase due to the Nowruz holidays within these periods

References

1. Iran population. https://knoema.com/atlas/Iran/Population. Accessed April 10, 2020.
2. Statistical Centre of Iran. *Selected Findings of the 2016 National Population and Housing Census*.; 2018.

https://www.amar.org.ir/Portals/1/census/2016/Census_2016_Selected_Findings.pdf. Accessed April 11, 2020.

3. Wan H, Cui J, Yang G-J. Risk estimation and prediction by modeling the transmission of the novel coronavirus (COVID-19) in mainland China excluding Hubei province. *medRxiv*. 2020.
4. Burke RM, Midgley CM, Dratch A, et al. Active Monitoring of Persons Exposed to Patients with Confirmed COVID-19 - United States, January-February 2020. *MMWR Morb Mortal Wkly Rep*. 2020;69(9):245-246. doi:10.15585/mmwr.mm6909e1
5. Haghdoost AA, Gooya MM, Baneshi MR. Modelling of H1N1 flu in Iran. 2009.
6. You C, Deng Y, Hu W, et al. Estimation of the time-varying reproduction number of COVID-19 outbreak in China. *Available SSRN 3539694*. 2020.
7. Mossong J, Hens N, Jit M, et al. Social contacts and mixing patterns relevant to the spread of infectious diseases. *PLoS Med*. 2008;5(3):0381-0391. doi:10.1371/journal.pmed.0050074
8. Ibuka Y, Ohkusa Y, Sugawara T, et al. Social contacts, vaccination decisions and influenza in Japan. *J Epidemiol Community Heal*. 2016;70(2):162-167. doi:10.1136/jech-2015-205777
9. Tang B, Bragazzi NL, Li Q, Tang S, Xiao Y, Wu J. An updated estimation of the risk of transmission of the novel coronavirus (2019-nCov). *Infect Dis Model*. 2020;5:248-255. doi:10.1016/j.idm.2020.02.001
10. Arregui S, Aleta A, Sanz J, Moreno Y. Projecting social contact matrices to different demographic structures. *PLoS Comput Biol*. 2018;14(12):e1006638. doi:10.1371/journal.pcbi.1006638
11. Hoang T, Coletti P, Melegaro A, et al. A systematic review of social contact surveys to inform transmission models of close-contact infections. *Epidemiology*. 2019;30(5):723-736.
