# Supplementary material for: Estimating COVID-19-Related Infections, Deaths, and Hospitalizations in Iran Under Different Physical Distancing and Isolation Scenarios
Source: Int J Health Policy Manag. 2020 Aug 1;11(3):334–43. doi: 10.34172/ijhpm.2020.134 (PMC9278464; doi:10.34172/ijhpm.2020.134)
Supplement: Supplementary file 3 — Calculation of Reproduction Number (R0). [file ijhpm-11-334-s003.pdf]

Supplementary file 3. Calculation of Reproduction Number (R_0)

We calculated the reproduction number using this formula[1]:

Eq 9:

$$\frac{dS(t)}{dt} = -\beta(t)C(t)\frac{II(t)}{N}S(t)$$
$$\frac{dE(t)}{dt} = \beta(t)C(t)\frac{II(t)}{N}S(t) - \frac{1}{D1}E(t)$$
$$\frac{dI(t)}{dt} = \frac{1}{D1}E(t) - \left(\frac{I.Is.R}{D6} + \frac{I.R.R}{D8} + \frac{IH.R}{D2} + \frac{ID.R}{D9}\right)I(t)$$
$$\frac{dR(t)}{dt} = \frac{TR.R}{D5}T(t) + \frac{I.R.R}{D8}I(t) + \frac{1}{D7}Is(t)$$
$$\frac{dIs(t)}{dt} = \frac{I.Is.R}{D6}I(t) - \frac{1}{D7}Is(t)$$
$$\frac{dH(t)}{dt} = \frac{IH.R}{D2}I(t) - \left(\frac{HD.R}{D3} + \frac{HT.R}{D4}\right)H(t)$$
$$\frac{dT(t)}{dt} = \frac{HT.R}{D4}H(t) - \left(\frac{TR.R}{D5} + \frac{TD.R}{D10}\right)T(t)$$
$$\frac{dD(t)}{dt} = \frac{\mathrm{HD.R}}{\mathrm{D3}}\mathrm{H(t)} + \frac{\mathrm{ID.R}}{\mathrm{D9}}\mathrm{I(t)} + \frac{TD.R}{D10}T(t)$$

$$F(X)=\begin{pmatrix} \beta(t)C(t)\frac{II(t)}{N} \\ 0 \\ 0 \\ 0 \\ 0 \\ 0 \\ 0 \end{pmatrix} \qquad V(X)=\begin{pmatrix} \frac{1}{D1}E(t) \\ -\frac{1}{D1}E(t) + \left(\frac{I.Is.R}{D6} + \frac{I.R.R}{D8} + \frac{IH.R}{D2} + \frac{ID.R}{D9}\right)I(t) \\ -\frac{TR.R}{D5}T(t) - \frac{I.R.R}{D8}I(t) - \frac{1}{D7} \\ -\frac{I.Is.R}{D6}I(t) + \frac{1}{D7}Is(t) \\ -\frac{IH.R}{D2}I(t) + \left(\frac{HD.R}{D3} + \frac{HT.R}{D4}\right)H(t) \\ -\frac{HT.R}{D4}H(t) + \left(\frac{TR.R}{D5} + \frac{TD.R}{D10}\right)T(t) \\ -\frac{HD.R}{D3}H(t) - \frac{ID.R}{D9}I(t) - \frac{TD.R}{D10}T(t) \end{pmatrix}$$

The Jacobian matrices of F(X) and V(X) at the disease-free equilibrium X_0 respectively,

DFE F(X) = $\begin{pmatrix} F & 0 \\ 0 & 0 \end{pmatrix}$

DFE V(X) = $\begin{pmatrix} V & 0 \\ J_1 & J_2 \end{pmatrix}$

Then,

F=$\begin{pmatrix} 0 & \beta(t) * C(t) \\ 0 & 0 \end{pmatrix}$

$$V=\begin{pmatrix} \frac{1}{D1} & 0 \\ -\frac{1}{D1} & \left(\frac{I.Is.R}{D6}+\frac{I.R.R}{D8}+\frac{IH.R}{D2}+\frac{ID.R}{D9}\right) \end{pmatrix}$$

We calculated V^{-1} and FV^{-1} and R_0

$$V^{-1}=\frac{1}{\frac{1}{D1}*\left(\frac{I.Is.R}{D6}+\frac{I.R.R}{D8}+\frac{IH.R}{D2}+\frac{ID.R}{D9}\right)}\begin{pmatrix} \left(\frac{I.Is.R}{D6}+\frac{I.R.R}{D8}+\frac{IH.R}{D2}+\frac{ID.R}{D9}\right) & 0 \\ \frac{1}{D1} & \frac{1}{D1} \end{pmatrix}$$

$$FV^{-1}=\begin{pmatrix} \frac{\beta(t)*C(t)}{\left(\frac{I.Is.R}{D6}+\frac{I.R.R}{D8}+\frac{IH.R}{D2}+\frac{ID.R}{D9}\right)} & \frac{\beta(t)*C(t)}{\left(\frac{I.Is.R}{D6}+\frac{I.R.R}{D8}+\frac{IH.R}{D2}+\frac{ID.R}{D9}\right)} \\ 0 & 0 \end{pmatrix}$$

$$\rho(FV^{-1})=\frac{\beta(t)*C(t)}{\left(\frac{I.Is.R}{D6}+\frac{I.R.R}{D8}+\frac{IH.R}{D2}+\frac{ID.R}{D9}\right)}$$

$$R_e=\frac{\beta(t)*C(t)}{\left(\frac{I.Is.R}{D6}+\frac{I.R.R}{D8}+\frac{IH.R}{D2}+\frac{ID.R}{D9}\right)}$$

References

1. Van den Driessche P, Watmough J. Reproduction numbers and sub-threshold endemic equilibria for compartmental models of disease transmission. *Math Biosci*. 2002;180(1-2):29-48. doi: 10.1016/S0025-5564(02)00108-6
